# TFPI Alpha and Beta Regulate mRNAs and microRNAs Involved in Cancer Biology and in the Immune System in Breast Cancer Cells

**DOI:** 10.1371/journal.pone.0047184

**Published:** 2012-10-05

**Authors:** Benedicte Stavik, Grethe Skretting, Ole Kristoffer Olstad, Marit Sletten, Magnus Dehli Vigeland, Per Morten Sandset, Nina Iversen

**Affiliations:** 1 Department of Medical Genetics, Oslo University Hospital and University of Oslo, Oslo, Norway; 2 Department of Haematology and Research Institute of Internal Medicine, Oslo University Hospital, Rikshospitalet, Oslo, Norway; 3 Department of Medical Biochemistry, Oslo University Hospital, Oslo, Norway; 4 Institute of Clinical Medicine, University of Oslo, Oslo, Norway; University of Bergen, Norway

## Abstract

Emerging evidence indicate a new role of TFPI in cancer biology. We recently reported that both isoforms of TFPI induced apoptosis and inhibited proliferation of cancer cells. The signaling pathway(s) mediating the effects of TFPI is, however, presently still unclear. Our goal was to further investigate the cellular processes affected by TFPI and to get insight into the molecular mechanisms involved in the effects of TFPI, using a global gene expression study approach. TFPIα or TFPIβ cDNA were transfected into SK-BR-3 breast cancer cells for stable overexpression. Global mRNA and microRNA (miRNA) expressions were measured and functional annotation of the differentially expressed genes and miRNAs according to gene ontology terms was conducted. Selected results were validated using qRT-PCR and Western blot. A total of 242 and 801 mRNA transcripts and 120 and 46 miRNAs were differentially expressed in cells overexpressing TFPIα or TFPIβ, respectively. Overexpression of either isoform significantly affected the expression of genes involved in cell development (apoptosis, cell movement, migration, invasion, colony formation, growth, and adhesion) and immune response. Network analyses revealed biological interactions between these genes and implied that several of the genes may be involved in both processes. The expression profiles also correlated significantly with clinical phenotype and outcome. Functional cluster analyses indicated altered activity of the epidermal growth factor receptor, small GTPases, and the NF-κB and JAK/STAT cascades when TFPI was overexpressed, and increased activity of the transcription factors NF-κB and Elk-1 and phospho-Akt levels was observed. Integrated mRNA-miRNA analyses showed that 19% and 32% of the differentially expressed genes in cells overexpressing TFPIα or TFPIβ, respectively, may have been regulated by miRNAs. Overexpression of TFPI in breast cancer cells affected the expression of mRNAs and miRNAs involved in processes facilitating cancer cell growth and immunologic response, possibly by signal transduction involving the EGFR pathway.

## Introduction

Tissue factor (TF) pathway inhibitor-1 (TFPI) is a serine protease inhibitor encoded on chromosome 2. Alternative splicing of the TFPI gene results in two main isoforms, TFPIα and TFPIβ. The 276 amino acid TFPIα contains three Kunitz protease inhibitor domains and a basic C-terminal end [1]. It is secreted from cells, and found either free in the extracellular compartment or bound to the cell membrane through a yet unidentified glycosyl-phosphatidylinositol (GPI) anchored protein [2]. TFPIβ consists of 223 amino acids, sharing amino acids 1–181 with TFPIα, and thus contains the first two Kunitz protease inhibitor domains. The C-terminal end encodes a GPI anchor attachment site [2], and TFPIβ is therefore located exclusively on the cell surface.

Microarrays are widely used for the simultaneous screening of whole genome mRNA expression, giving extensive information about the transcriptome. Functional analysis of expression signatures elucidates ongoing cellular and molecular processes. Expression profiles derived from clinically assessed breast tumors also aid in tumor classification and prognostic assessment [3]. MicroRNAs (miRNAs) are short (∼22 nt) non-coding RNA fragments that regulate mRNA expression at the post-transcriptional level. Since their discovery in 1993 [4], more than 1500 human miRNAs have been identified according to the miRNA sequence database (http://www.mirbase.org/, release 18). Through partial sequence complementarity, miRNAs bind to the 3′ untranslated region (UTR) of their target mRNAs and facilitate cleavage or degradation of the transcripts [5], [6]. Many miRNAs are known to be important in relation to disease, such as cancer, as they regulate genes involved in proliferation, differentiation, and apoptosis [5], [7], [8]. Altered expression of miRNAs has been observed in human breast cancers, and several of the miRNAs have been shown to regulate tumorigenic processes [9]–[13].

In general, TFPI is known for its important role in the regulation of TF induced blood coagulation. However, more recent evidence indicates an additional role of TFPI in cancer. Several cancer tissues and cell lines have been shown to express TFPI [14], [15], and TFPI treatment has been reported to reduce tumor growth and metastasis *in vivo* [16], [17]. We recently reported that overexpression of TFPIα or TFPIβ in SK-BR-3 breast cancer cells resulted in pro-apoptotic and anti-proliferative effects of either isoform *in vitro* [18], but the mechanism behind the effect of TFPI is poorly understood.

In the present study, the global mRNA expression profiles of SK-BR-3 breast cancer cells stably overexpressing TFPIα or TFPIβ were investigated to further understand the cellular processes affected beyond apoptosis and proliferation, and the molecular mechanisms behind these effects. The clinical relevance of the differentially expressed genes was assessed using publicly available, clinically annotated breast cancer expression data. To our knowledge, no reports have described the relationship between TFPI and miRNA expressions. A miRNA screening was therefore conducted to elucidate the possible involvement of these mRNA regulators in mediating the cellular effects of TFPI.

## Materials and Methods

### Cell cultures and stable cell lines

The human mammary adenocarcinoma SK-BR-3 cells (ATCC HTB-30, Manassas, VA, USA) were grown in RPMI1640 containing phenol red and 2 mM L-glutamine (Lonza, Viviere, Belgium), supplemented with 10% heat inactivated FBS (Lonza). Cells were cultured at 37°C in an incubator with a humidified atmosphere and 5% CO2.

Stable cell lines with TFPI upregulated were established as previously described [18]. In short, cDNAs encoding TFPIα or TFPIβ were cloned into the pcDNA3.1/V5/His-pTOPO vector (Invitrogen Life Technology, Carlsbad, CA) and transfected into SK-BR-3 wild type cells using Lipofectamine 2000. Stable clones were selected using neomycin. The stable cell line pools with TFPIα or TFPIβ upregulated were named pTOPO-TFPIα and pTOPO-TFPIβ, respectively. To minimize non-specific effects of the stable overexpression, wild type cells transfected with an empty vector pTOPO were used as controls.

### RNA isolation

Total RNA was isolated from the cells using the mirVANA RNA isolation kit (Ambion Life Technologies) according to the manufacturer's instruction. The quantity and quality of the isolated RNA were measured using the NanoDrop® ND-1000 UV-Vis Spectrophotometer (NanoDrop Technologies, Wilmington, DE) and Agilent 2100 Bioanalyzer (Agilent Technologies, Palo Alto, CA), respectively.

### Microarray analyses and statistical analysis of data

Microarray analyses were performed using the Affymetrix GeneChip Human Gene 1.0 ST Arrays (Affymetrix, Santa Clara, CA) which contains approximately 28,000 gene transcripts. 150 ng of total RNA was subjected to GeneChip HT One-Cycle cDNA Synthesis Kit and GeneChip HT IVT Labeling Kit, following the manufacturer's protocol for whole genome gene expression analysis (Affymetrix). Biotinylated and fragmented single stranded cDNAs were hybridized to the GeneChips. The arrays were washed and stained using FS-450 fluidics station (Affymetrix). Signal intensities were detected by Hewlett Packard Gene Array Scanner 3000 7G (Hewlett Packard, Palo Alto, CA, USA). Three chips with samples from three independent RNA isolations were run for each cell line.

The scanned images were processed using the AGCC (Affymetrix GeneChip Command Console) software and the CEL files were imported into the Partek Genomics Suite software (Partek, Inc. MO, USA). The Robust Multichip Analysis (RMA) algorithm was applied for generation of signal values and normalization. Probe sets with maximal signal values of less than 5 across all arrays were removed to filter for low and non-expressed genes, reducing the number of mRNA transcripts to 25,492. For expression comparisons of different groups, profiles were compared using a 1-way ANOVA model. The results were expressed as fold changes (FC), i.e., ratios of mean signal values from cells with TFPI upregulated and empty vector control cells. Gene lists were generated with the criteria of false discovery rate (FDR) 10% and a FC of ≥|2|. The data has been deposited in the NCBI Gene Expression Omnibus (GEO) database with accession number GSE30037 in compliance to MIAME guidelines (http://www.ncbi.nlm.nih.gov/geo/).

### Analysis of clinical breast cancer datasets

The publicly available breast cancer datasets GSE6532, GSE4922, and GSE7390 were downloaded from GEO at NCBI and merged. Expression data generated from untreated patients using the Affymetrix HG-U133A arrays were selected, and profiles from samples not containing the clinical variables age, size, grade, estrogen receptor status, relapse-free survival time, and relapse-free survival were excluded, resulting in 545 samples. The datasets were RMA normalized individually using the R/Bioconductor package *affy*, and each probe median centered before merging. The probe IDs of the 242 and 801 differentially expressed genes following overexpression of TFPIα or TFPIβ, respectively, were matched to probe IDs identical to the IDs used in the clinical datasets using netaffx, giving 262 and 773 probes available for comparison. Only these probes were extracted from the merged dataset and used in the analysis. Associations between the differentially expressed genes following overexpression of either isoform of TFPI and the clinical gene profiles were evaluated using the *globaltest* package in R. In each test of a clinical variable, all other clinical variables were controlled for. The resulting *p*-values were adjusted for multiple testing with Hommel's method in R.

The correlation between the expression of TFPI isoforms in the normalized datasets and the clinical variables were investigated using a non-parametric Spearman's rho correlation test in R. *P*-values were corrected using the Holm-Bonferroni method.

### MicroRNA profiling and target gene prediction

miRNA quantification was conducted using the Taqman Human MicroRNA Array Card A v2.0 (Applied Biosystems Life Technologies) following the manufacturer's instructions. The array measures the expression of 377 highly characterized miRNAs and 4 controls simultaneously. In short, 350 ng total RNA was reversely transcribed into cDNA using the Megaplex Human RT primers pool A and TaqMan MicroRNA RT kit (Applied Biosystems Life Technologies). After addition of TaqMan Gene Expression Master mix (Applied Biosystems Life Technologies), samples were loaded on the arrays and run on the ABI PRISM 7900 HT Sequence Detection System (Applied Biosystems Life Technologies) using the low density array format. Results were normalized against the endogenous control U6 snRNA and changes in relative miRNA expression were calculated using the comparative Ct method and expressed as FC of empty vector pTOPO control. miRNAs with a FC of ≥|2| were considered differentially expressed.

For the integrated miRNA and mRNA analysis, the lists of differentially expressed miRNAs were loaded into the Partek Genomics Suite software (Partek) already containing the analyzed microarray data. The software connects to the targetscan 5.1 database (http://www.targetscan.org/) for identification of predicted mRNAs possible regulated by the differentially expressed miRNAs.

### qRT-PCR validation of microarray and miRNA results

Quantitative real-time PCR (qRT-PCR) was performed on selected mRNAs and miRNAs using Taqman single assays (Applied Biosystems Life Technology) to verify the array expression results. For validation of the mRNA data, samples were prepared as previously reported [18] using assays detecting ASCL1 (Hs00269932_m1), BCL2 (Hs00608023_m1), EGFR (Hs01076076_m1), ERBB2 (Hs01001580_m1), DOCK11 (Hs00376176_m1), GPNMB (Hs01095679_m1), IFI27 (Hs00271467_m1), IFIT2 (fwd primer: 5'- CCCAGCATCAGCCACACT-‘3, rev primer: 5’- AAAAACCATGAATTTGTATTGTTTTAATTGCACAA-‘3, reporter: 5’- CTGGGTTGGAAAATGT-‘3), IFITM1 (fwd primer: 5’- GGCTCTGTGACAGTCTACCATATT-‘3, rev primer: 5’- GCTATGGGCGGCTACTAGTAAC-‘3, reporter: 5’- CCCGTTTTTCCTGTATTATC-‘3), IGF1R (Hs00609566_m1), MX1 (fwd primer: 5’- TGCTGAACATCACAGCTTATTTCCT-‘3, rev primer: 5’- CGGCACTCATGCTCCTAAAACA-‘3, reporter: 5’- CTGGGTTTGTGAAGGGACAT-‘3), NFKBIA (Hs00153283_m1), PGR (Hs01556702_m1), RET (Hs01120030_m1), SERPINE1 (Hs00167155_m1), and STAT3 (Hs00234174_m1). For validation of the miRNA results, samples were prepared using the TaqMan MicroRNA RT kit (Applied Biosystems Life Technologies) as described by the manufacturer with assays detecting let-7d (#002283), miR-103 (#000439), miR-106b (#000442), miR-126 (#002228), miR-135a (#000460), miR-135b (#002261), miR-21 (#000397), miR-29c (#000587), miR-205 (#000509), miR-222 (#002276), miR-342-3p (#002260), miR-365 (#001020), and miR-652 (#002352). All samples were run in triplicates on the ABI PRISM 7900 HT Sequence Detection System (Applied Biosystems Life Technologies). mRNA and miRNA results were normalized against the endogenous control PMM1 (Hs00160195_m1) or U6 snRNA (#001973), respectively, and changes in relative expression were calculated using the comparative Ct method, as described in User Bulletin No.2 ABI Prism 7700 Sequence Detection System (Applied Biosystems Life Technologies). The correlations between the array and qRT-PCR measurements were statistically assessed using the Spearman non-parametric correlation test in Graphpad Prism 5.0 (Graphpad, San Diego, CA, USA).

### Western blot analysis

For Western blot analysis, cells were harvested and lysed as described previously [18]. The cell lysates were incubated at 97°C for 5 minutes before separated on a SDS-polyacrylamide gel (Bio-Rad Laboratories, Hercules, CA). The membrane was incubated over night at 4°C with the primary human antibodies anti-phospho-Akt, anti-Akt (#9271 and #9272, Cell Signaling Technology, Denver MA), anti-EGFR (#44–798G, Invitrogen Life Technologies) and anti-α-tubulin (#T5168, Sigma-Aldrich, St. Louis, MO) and for 1 hour at room temperature with the appropriate horseradish peroxidase (HRP) -conjugated secondary antibody. HRP signals were developed using the ECL Western blotting detection system (GE Healthcare, Fairfield, CT). Bands were quantified using the image processing and analysis program ImageJ version 1.4.3.67 (Rasband W; National Institute of Health, Bethesda, MD, http://rsbweb.nih.gov/ij/index.html).

### Luciferase analysis

Pathway analysis was performed using Cignal Finder^TM^ 10-Pathway Reporter Arrays (SuperArray Biosciences, Fredrick, MD) according to the manufacturer's instruction. In short, SK-BR-3 cells (3×10^4^) transiently overexpressing TFPIα or TFPIβ or the empty vector pTOPO as a control were seeded into 96-well arrays containing luciferase reporters to common signal transduction pathways and transfection agent. After 48 h, cells were lysed and luciferase activity measured using a Wallac Victor 1420 plate-reader (Perkin Elmer, Waltham, MA). The intensity of the firefly luciferase was normalized to renilla luciferase, and the firefly/renilla ratios of cells overexpressing TFPI and empty vector control cells were divided to determine the relative luciferase activity.

## Results

### Global changes in mRNA expression

The stable overexpression of the two isoforms of TFPI in SK-BR-3 breast cancer cells have previously been reported [18] and are summarized in Table 1. TFPIβ is exclusively located on the cell surface and an increase in protein expression was therefore only detected in the cell medium after PI-PLC treatment, which cleaves the GPI anchor and releases TFPIβ from the surface. The effect of the upregulation of TFPIα or TFPIβ on the global mRNA expression was investigated using microarray technology. After correcting for multiple testing, only genes with a FC of ≥|2| and a *P*-value of >.05 were considered differentially expressed. A total of 242 transcripts were found to be differentially expressed in SK-BR-3 cells overexpressing TFPIα compared to empty vector control cells. 223 of these were upregulated (top ten: *IFI44, IFI44L, IFIT1, MX1, SAMD9, DDX60, IFIT3, OAS2, IFIT2, PARP9*), while 19 were downregulated (top ten: *ASCL1, RET, SBCB, SLC4A10, PODXL, SLC6A14, FREM2, IGF1R, LXN, RN5S9*; Table S1). In comparison, 801 transcripts were differentially expressed in SK-BR-3 cells with TFPIβ overexpressed. Of these, 546 (top ten: *GPNMB, WNT5A, DOCK11, SCIN, PLS3, KRT4, VGLL1, LDHB, GJA5, SCL15A1*) and 175 (top ten: *ASCL1, NCRNA00052, SLITRK6, PCDH10, NPY1R, NCAM2, PGR, C6orf141, SGCG, RET*) were up- or downregulated, respectively, compared to control cells (Table S2). 106 of the differentially expressed mRNAs were identified in both cell lines overexpressing TFPI.

**Table 1:** Overexpression of TFPI in breast cancer cells.

		TFPI mRNA FC of control	Total TFPI ag FC of control
SK-BR-3	pTOPO-TFPIα	34±2.1	6.4±0.1
	pTOPO-TFPIβ	55±6.4	17.3±0.1*
	pTOPO (control)	1±0.1	1.0±0.1

Overexpression of TFPIα or TFPIβ in SK-BR-3 breast cancer cells, displayed as a summary of previously reported data [18]. mRNA levels were quantified using qRT-PCR. Protein levels were measured in cell medium using total TFPI ELISA. Results are presented relatively as mean fold change (FC) of control (n = 3± SD). * Relative increase in TFPI antigen measured in cell medium after PI-PLC treatment.

### Gene ontology categories

The results generated from the microarray experiments were loaded into the Ingenuity Pathway Analysis software (Ingenuity Systems, www.ingenuity.com) for functional analysis. 183 and 669 of the transcript IDs that were differentially expressed in cells overexpressing TFPIα or TFPIβ, respectively, were mapped in the ingenuity analysis. Of these, 158 and 534 were eligible for functional analysis. The ten most significant biological functions affected are listed in Table 2 (for complete lists, see Table S3). Eight of the ten gene ontology (GO) categories, Cell Death, Cellular Development, Cellular Movement, Cellular Growth and Proliferation, Cell-To-Cell Signaling and Interaction, Cellular Function and Maintenance, Cell Morphology, and Cellular Assembly and Organization, were affected when either isoform was overexpressed. Within these categories, lower GO terms such as apoptosis, cell movement, migration, invasion, colony formation, proliferation, and adhesion were affected. The ten most significant diseases are listed in Table 2 (For complete lists, see Table S3). Four of these, Infectious Disease, Cancer, Inflammatory Disease, and Reproductive System Disease, were identified in both cell lines. Within the cancer category, lower GO terms such as tumorigenesis and metastasis were affected. We then investigated the differentially expressed genes associated with the lower GO terms. Several genes were found to be associated with more than one category and some seemed to play key roles. The key genes included *AQP3*, *ASCL1*, *BAK1*, *CAPN2*, *CASP4*, *CDK12*, *CTSC*, *CYP1B1*, *DOCK11*, *EGFR*, *EPAS1*, *FERMT2*, *GLRX*, *IFIH1*, *IGF1R*, *ITGB6*, *LGALS3BP*, *MED1*, *MYDD88*, *NAV1*, *NT5E*, *NTN4*, *OLR1*, *PDGFD*, *PODXL*, *RET*, *SCIN*, *SGCG*, *SCL15A1*, *ST6GAL1*, *STAT3*, *SWAP70*, *TAP1*, *TIMP2*, *TPD52*, *TRIM29*, *VCL*, and *WNT5A* when either isoform of TFPI was overexpressed. The differentially expressed genes associated with Inflammatory disease were also investigated and in addition to the underlined genes listed above, *FREM2*, *KIAA1217*, *OSBPL3*, *PSMB8*, *RIN2*, *SLC41A2*, *UNC93B1*, and *WBSCR17* were identified in both cell lines.

**Table 2:** Functional annotation of differentially expressed mRNAs following overexpression of TFPIα or TFPIβ.

TFPIα vs.pTOPO Gene ontology category	Significance	# Genes	TFPIβ vs. pTOPO Gene ontology category	Significance	# Genes
***Molecular and cellular function***			***Molecular and cellular function***		
Gene Expression	1.20E-07-9.31E-03	37	Cellular Growth and Proliferation	9.92E-08-1.92E-02	109
Cell Death	1.20E-06-1.07E-02	64	Cellular Movement	1.14E-07-1.93E-02	97
Cellular Development	3.41E-06-1.09E-02	47	Carbohydrate Metabolism	2.16E-05-1.83E-02	21
Cellular Movement	1.05E-05-7.82E-03	38	Cell Death	2.72E-05-1.86E-02	133
Cellular Growth and Proliferation	1.13E-05-1.07E-02	52	Cell-To-Cell Signaling and Interaction	6.18E-05-1.61E-02	81
Cell-To-Cell Signaling and Interaction	3.40E-05-1.09E-02	45	Cell Morphology	6.38E-05-1.92E-02	79
Cellular Function and Maintenance	1.49E-04-1.09E-02	26	Cellular Assembly and Organization	6.38E-05-1.92E-02	72
Cell Morphology	1.53E-04-1.09E-02	17	Cellular Function and Maintenance	1.23E-04-1.97E-02	45
Cellular Assembly and Organization	1.53E-04-4.41E-03	12	Protein Synthesis	1.23E-04-1.23E-04	9
Post-Translational Modification	1.53E-04-8.71E-04	6	Cellular Development	1.46E-04-1.92E-02	95
***Diseases and disorders***			***Diseases and disorders***		
Infection Mechanism	3.56E-16-9.31E-03	44	Genetic Disorder	1.35E-07-1.02E-02	361
Antimicrobial Response	2.00E-14-3.09E-03	17	Cancer	1.40E-05-1.92E-02	183
Inflammatory Response	2.00E-14-8.98E-03	56	Reproductive System Disease	1.40E-05-1.53E-02	92
Organismal Injury and Abnormalities	9.91E-14-5.96E-03	39	Cardiovascular Disease	2.19E-05-1.87E-02	143
Infectious Disease	3.36E-07-1.04E-02	43	Endocrine System Disorders	2.54E-04-1.70E-03	144
Immunological Disease	6.84E-07-9.31E-03	55	Metabolic Disease	2.54E-04-1.70E-03	156
Cancer	1.22E-06-1.06E-02	70	Renal and Urological Disease	3.79E-04-4.58E-03	7
Inflammatory Disease	2.05E-06-7.71E-03	67	Gastrointestinal Disease	3.84E-04-1.92E-02	90
Reproductive System Disease	3.17E-06-5.96E-03	19	Inflammatory Disease	3.84E-04-1.64E-02	168
Hepatic System Disease	7.15E-05-7.82E-03	29	Infectious Disease	6.89E-04-9.96E-03	6

The ten most significant categories involved in molecular and cellular function and diseases and disorders, as annotated by the Ingenuity Pathway Analysis software, are listed.

### Network analysis

To investigate the biological interaction between the differentially expressed genes we generated networks using the Ingenuity Pathway Analysis software. Of the 183 and 669 transcript IDs mapped in the ingenuity analyses, 161 and 560 were network eligible. To express the probability that the genes in a network are actually connected, a *p*-value is calculated and displayed as the -log value (z-score). All the interactions were species specific. Overexpression of TFPIα resulted in ten networks with a z-score of >15 (*p*<1.0e^−15^, Table S4). The networks included genes involved in the immune system, cell death, protein modification, gene expression, cell communication, cell movement and cancer. Networks one to nine overlapped and several of the genes were connected to more than one network. The most significant network associated with both biological function and disease contained genes involved in Inflammatory Response, Infection Mechanism, and Cell Death. Two genes in this network, *BAK1* and *TNFSF10*, were found to be associated with both immune response and cell death (Figure S1). Overexpression of TFPIβ resulted in 16 networks with a z-score of >15 (Table S4). These networks included genes involved in metabolism, growth, immune system, cardiovascular system, cell movement, cell development, cell cycle, gene expression, cancer, cellular organization and cell death. Seven of these networks (network 1, 2, 7–9, 13 and 14) overlapped, and there were some interactions between the networks. The most significant network associated with both biological function and disease contained genes involved in Cellular Growth and Proliferation, Inflammatory Disease, and Respiratory Disease. Five genes, *EGFR*, *NCOA3*, *PRKAR2B*, *SAT1* and *STAT3* were associated with both inflammatory disorder and cellular growth in this network (Figure S2).

### Signal transduction mechanisms

To explore the candidate signal transduction mechanisms possibly mediating the effects of TFPI, we investigated the functional clusters among the differentially expressed genes using eGOn v2.0 (http://www.genetools.microarray.ntnu.no/egon/index.php). The Affymetrix IDs were converted to Genebank accession numbers using DAVID v6.7 (http://david.abcc.ncifcrf.gov/) and of the 242 and 801 transcripts with Affymetrix IDs, 222 and 753 were converted to Genebank accession numbers and loaded into eGOn. 207 and 687 transcripts were mapped to unigene clusters and 156 and 554 of these were associated with GO terms when TFPIα or TFPIβ was overexpressed, respectively. The list of all the expressed genes contained 25,492 transcripts IDs, of which 24,371 were converted to Unigene IDs and uploaded. 23,332 were mapped to unigene clusters and 13,061 were associated with GO terms. Using the Master-Target test, the differentially expressed genes identified in cells overexpressing TFPIα or TFPIβ were compared to all the expressed genes in the microarray analysis to distinguish GO categories significantly over-represented. Results showed a significant enrichment of genes involved in the NFκB (*BST2*, *LGALS9*, *NFKBIA*, *STAT1*, *TNFSF10*, *TRIM38*) and JAK/STAT (*NMI*, *STAT1*, *STAT2*, *STAT3*) signaling pathway when TFPIα was overexpressed (Table 3). In cells overexpressing TFPIβ, a general effect on the intracellular signaling and receptor tyrosine kinase activity (*EGFR*, *EPHA4*, *EHPA7*, *ERBB2*, *FGFR2*, *RET*, *TYRO3*) was observed (Table 3). Epidermal growth factor receptor (EGFR) activity (*EGFR*, *ERBB2*) and small GTPase signaling (*ARHGDIB*, *DIAPH3*, *DOCK3*, *DOCK4*, *DOCK9*, *DOCK11*, *ELMO1*, *EXPH5*, *GBP1*, *GBP3*, *GDI2*, *MX1*, *MX2*, *NRAS*, *PLCE1*, *RIN2*, *RND1*) were affected when either isoform was overexpressed.

**Table 3:** Signal transduction mechanisms affected after overexpression of TFPIα or TFPIβ.

GO number	Name	Whole array	TFPIα/β vs. pTOPO	*P*-value
**TFPIα vs. pTOPO**				
*GO:0008150*	*Biological process*	*10463*	*137*	
GO:0007243	protein kinase cascade	321	11	0.003
GO:0007249	I-kappaB kinase/NF-kappaB cascade	107	6	0.003
GO:0007253	cytoplasmic sequestering of NF-kappaB	3	1	0.039
GO:0007259	JAK-STAT cascade	31	4	0.001
GO:0008593	regulation of Notch signaling pathway	3	1	0.039
GO:0007186	G-protein coupled receptor protein signaling pathway	605	1	0.005
*GO:0003674*	*Molecular function*	11326	140	
GO:0005057	receptor signaling protein activity	119	6	0.004
GO:0005006	epidermal growth factor receptor activity	2	1	0.025
GO:0004694	eukaryotic translation initiation factor 2α kinase activity	3	1	0.037
GO:0004710	MAP/ERK kinase kinase activity	1	1	0.012
GO:0017112	Rab guanyl-nucleotide exchange factor activity	3	1	0.037
GO:0005093	Rab GDP-dissociation inhibitor activity	2	1	0.025
GO:0003924	GTPase activity	144	5	0.033
**TFPIβ vs. pTOPO**				
*GO:0008150*	*Biological process*	*10463*	*435*	
GO:0007242	intracellular signaling cascade	1142	63	0.018
GO:0042504	tyrosine phosphorylation of Stat4 protein	1	1	0.042
GO:0007173	epidermal growth factor receptor signaling pathway	25	5	0.003
GO:0016601	Rac protein signal transduction	9	3	0.005
GO:0034097	response to cytokine stimulus	3	2	0.005
GO:0030522	intracellular receptor-mediated signaling pathway	48	5	0.048
GO:0030518	steroid hormone receptor signaling pathway	42	5	0.029
GO:0030521	androgen receptor signaling pathway	28	4	0.027
*GO:0003674*	*Molecular function*	11326	482	
GO:0004714	transmembrane receptor protein tyrosine kinase activity	55	7	0.008
GO:0004710	MAP/ERK kinase kinase activity	1	1	0.043
GO:0008427	calcium-dependent protein kinase inhibitor activity	1	1	0.043
GO:0008083	growth factor activity	123	10	0.041
GO:0005006	epidermal growth factor receptor activity	2	2	0.002

The differentially expressed mRNAs were loaded into eGOn for functional annotation, and the Master-Target test was used to identify significantly over-represented gene ontology categories (by comparing the number of genes associated with a gene ontology category to the total number of genes in the array associated with that category).

**Figure 1:**
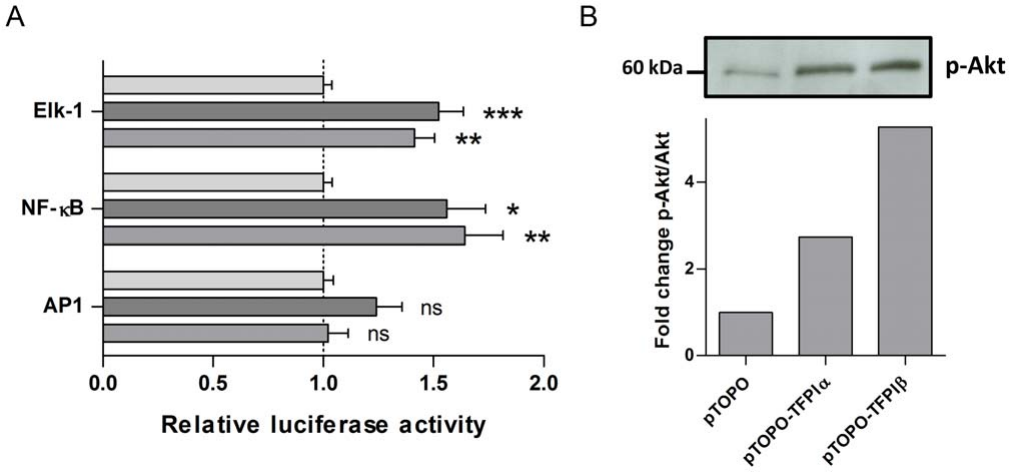
Transcription factor activity and phospho-Akt in cells after overexpression of TFPI. (A) Transcription factor activity was measured using the cignal finder luciferase reporter system. SK-BR-3 cells (3×10^4^) transiently transfected with vectors overexpressing TFPIα (dark gray) or TFPIβ (gray), or with empty vector pTOPO (light gray) as controls were seeded in 96-well arrays 24 hours after transfection. After 48 hours, cells were lysed and the firefly and renilla luciferase intensity determined. The results are presented as mean (n≥8) relative luciferase activity + SEM of three independent experiments. Statistical differences between cells overexpressing TFPI and empty vector control cells were determined using the student's t test (* *p*<.05, ** *p*<.01, *** *p*<.001, ns  =  not significant). (B) Western blot of phospho-Akt in SK-BR-3 cells overexpressing TFPI. Band intensities were measured using ImageJ and the intensities of phospho-Akt were normalized to Akt levels.

The luciferase reporter system cignal finder and western blotting were used to further identify the signaling molecules that were affected in cells overexpressing TFPI. Results showed that overexpression of either isoform of TFPI led to increased activity of the NF-κB and Elk-1 transcription factors, but not AP1 (Figure 1A). An increase in phospho-Akt was also observed in SK-BR-3 cells after overexpression of TFPIα or TFPIβ (Figure 1B).

### Clinical relevance of the differentially expressed genes

To further evaluate the involvement of TFPI in cancer progression, the clinical importance of the differentially expressed genes was assessed. Our data were compared to a merged dataset of publicly available, clinically annotated breast cancer expression profiles, comprised of 545 untreated patients. Results showed that the differentially expressed genes were significantly associated with tumor grade and ER status when either isoform of TFPI was overexpressed. Furthermore, overexpression of TFPIβ resulted in an expression signature that significantly correlated with relapse-free survival time and occurrence (Table 4).

**Table 4:** Clinical relevance of gene signatures following overexpression of TFPIα or TFPIβ.

Clinical Variable	TFPIα	TFPIβ
Merged Breast cancer datasets (n = 545)	*P*-value	*P*-value
**Age**	ns	4.96×10^−5^
**Tumor size**	ns	Ns
**Tumor grade**	4.87×10^−4^	2.41×10^−14^
**ER status**	1.09×10^−8^	2.04×10^−60^
**Relapse-free survival time**	ns	8.33×10^−4^
**Relapse-free survival**	ns	4.09×10^−4^

Three publicly available, clinically annotated breast cancer datasets (GSE6532, GSE4922, and GSE7390) were downloaded from the Gene Expression Omnibus (GEO) database at NCBI and merged. Associations between the differentially expressed genes and clinical variables were evaluated using the *globaltest* package in R.

We also investigated the correlation between the expression of TFPI isoforms and clinical variables in the merged datasets. The results showed that the expression of two probes identifying TFPIα (209676_at and 213258_at) correlated significantly with tumor size (*p* = .035 and .008, respectively).

### Changes in miRNA expression

**Figure 2:**
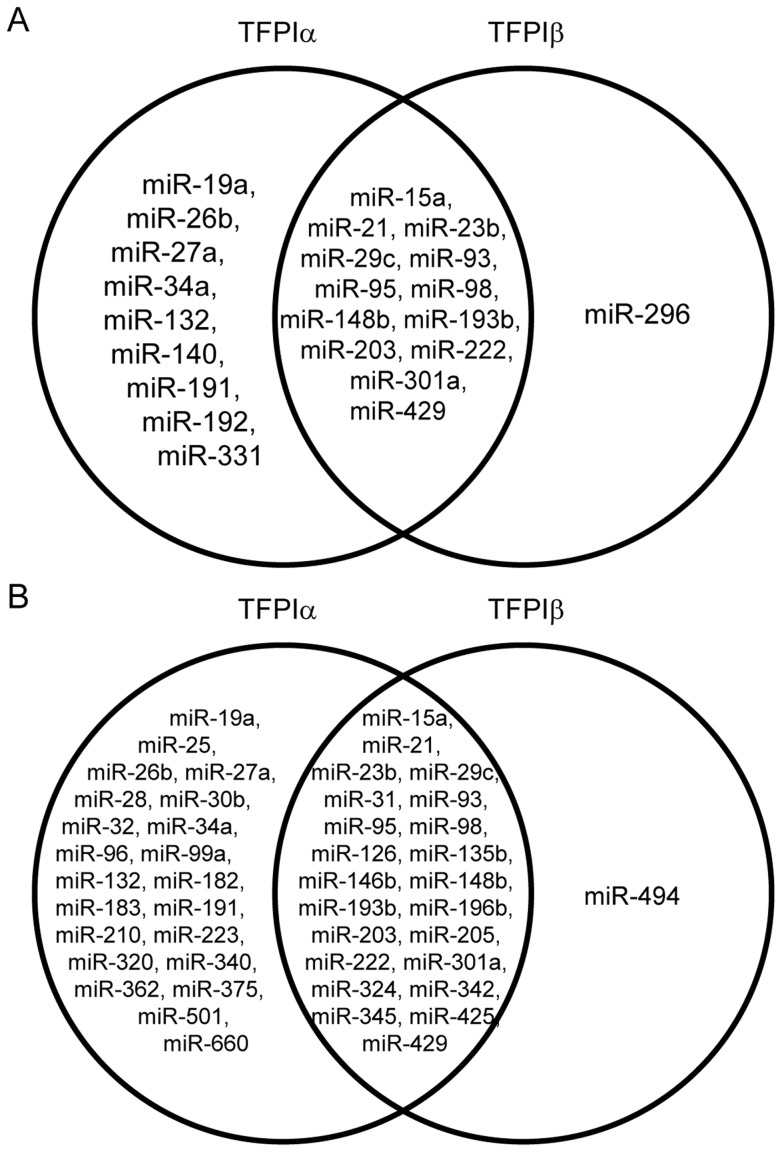
Venn diagrams of differentially expressed miRNAs after overexpression of TFPIα or TFPIβ. miRNAs associated with molecular and cellular function (A) and cancer disease (B), as annotated by the Ingenuity Pathway Analysis software.

We next investigated if overexpression of TFPI affected the expression of any miRNAs in the two cell lines. Overexpression of TFPIα resulted in differential expression of 120 different miRNAs, of which all were downregulated (top ten: miR-15a, miR-98, miR-628-5p, miR-128, miR-95, miR-340, miR-660, miR-9, miR-501-5p, miR-96; Table S5). In cells overexpressing TFPIβ, 46 miRNAs were differentially expressed; ten being upregulated (miR-23b, miR-205, miR-30c, miR-628-5p, miR-29c, miR-422a, miR130b, miR146b-5p, miR-184, miR-31) and 36 downregulated (top ten: miR-429, miR-95, miR-708, miR-200a, miR-126, miR-135a, miR-489, miR-218, miR-342-3p, miR-135b; Table S5). 34 of the differentially expressed miRNAs were identified in both cell lines overexpressing TFPI. The differentially expressed miRNAs were loaded into the Ingenuity Pathway Analysis software for functional analysis, and within biological function, GO categories such as Cellular Development, Cellular Growth and Proliferation, Cell Cycle, Cell-to-Cell Signaling and Interaction, and Cell Death were significantly affected (Table S6). Within these categories, 13 different miRNAs were differentially expressed in cells overexpressing either isoform of TFPI (Figure 2A). The disease associated GO categories Cancer and Inflammatory Disease were also significantly affected (Table S6), and 23 different miRNAs associated with these categories were differentially expressed in both cell lines (Figure 2B).

We investigated the possible interactions between the miRNA and mRNA expression results. Using the Partek software, the miRNA and mRNA data were compared to identify whether any of the predicted miRNA gene targets were actually differentially expressed at the mRNA level. The results showed that predicted targets of 83 miRNAs were oppositely expressed at the mRNA level in cells overexpressing TFPIα, possibly regulating 46 different mRNAs (Table S7). In cells overexpressing TFPIβ, predicted targets of 39 miRNAs were differentially expressed in the microarray analysis, possibly regulating 252 different mRNAs (Table S8). 26 miRNAs were identified in both cell lines overexpressing TFPI, with predicted target genes oppositely regulated. 16 of these had the same predicted targets oppositely expressed in both cell lines, which included *ACSL1*, *FERMT2*, *KIAA1217*, *KRT80*, *NAV1*, *NT5E*, *NTN4*, *PDGFD*, *RNF213*, *STAT3*, *SWAP70*, *TPD52*, and *WNT5A* (Table 5). All of these predicted targets except two (*RNF213* and *KRT80*) were among the key genes identified in the functional analysis above.

**Table 5:** Differentially expressed miRNAs with predicted mRNA targets oppositely regulated in both cell lines overexpressing TFPI.

miRNA	*Gene target(s)*	*Target names*	Target modulation (FC*)	
Downregulated microRNAs			TFPIα	TFPIβ
hsa-miR-15a	*NAV1*	neuron navigator 1	2.1	3.2
	*RNF213*	ring finger protein 213	2.2	2.3
	*SWAP70*	SWAP switching B-cell complex 70kDa subunit	2.2	2.9
hsa-miR-93	*NTN4*	netrin 4	2.6	3.8
	*STAT3*	signal transducer and activator of transcription 3	2.1	2.6
hsa-miR-101	*KIAA1217*		2.2	2.3
	*SWAP70*	SWAP switching B-cell complex 70kDa subunit	2.2	2.9
hsa-miR-135a	*FERMT2*	fermitin family member 2	2.4	2.6
hsa-miR-135b	*FERMT2*	fermitin family member 2	2.4	2.6
hsa-miR-148b	*KIAA1217*		2.2	2.3
hsa-miR-193b	*NT5E*	5'-nucleotidase, ecto (CD73)	2.6	2.3
hsa-miR-200a	*WNT5A*	wingless-type MMTV integration site family, member 5A	3.2	7.7
hsa-miR-200b	*KRT80*	keratin 80	2.2	2.0
hsa-miR-203	*ACSL1*	acyl-CoA synthetase long-chain family member 1	3.1	3.3
	*PDGFD*	platelet derived growth factor D	2.1	3.2
hsa-miR-218	*ACSL1*	acyl-CoA synthetase long-chain family member 1	3.1	3.3
	*TPD52*	tumor protein D52	2.0	2.5
hsa-miR-301a	*ACSL1*	acyl-CoA synthetase long-chain family member 1	3.1	3.3
	*FERMT2*	fermitin family member 2	2.4	2.6
	*KIAA1217*		2.2	2.3
hsa-miR-365	*WNT5A*	wingless-type MMTV integration site family, member 5A	3.2	7.7
hsa-miR-429	*KRT80*	keratin 80	2.2	2.0
hsa-miR-454	*ACSL1*	acyl-CoA synthetase long-chain family member 1	3.1	3.3
	*FERMT2*	fermitin family member 2	2.4	2.6
	*KIAA1217*		2.2	2.3
hsa-miR-636	*ACSL1*	acyl-CoA synthetase long-chain family member 1	3.1	3.3
	*TPD52*	tumor protein D52	2.0	2.5

* Fold change.

### qRT-PCR and Western blot validation

**Figure 3:**
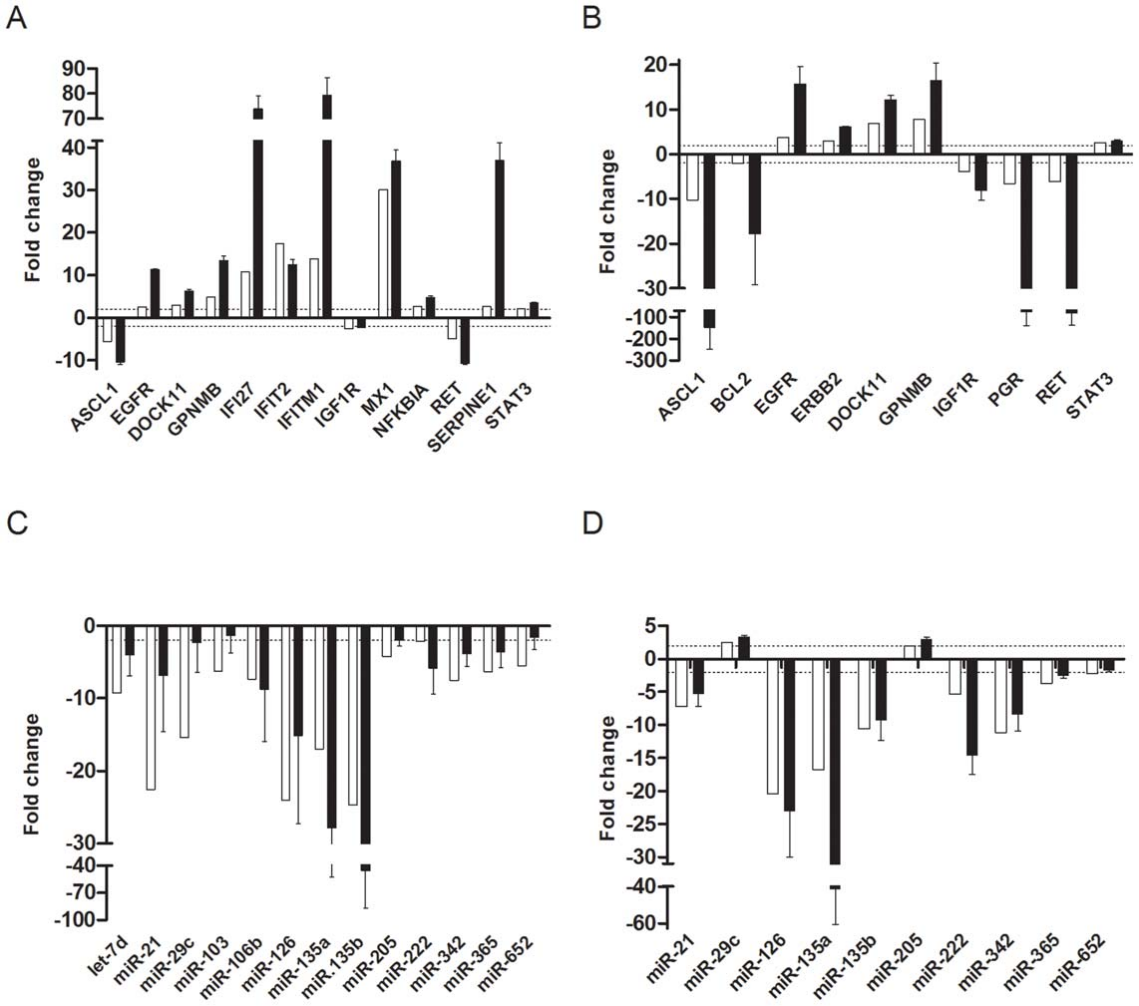
Validation of microarray and miRNA array expression results. Selected mRNAs (A and B) and miRNAs (C and D) differentially expressed in SK-BR-3 cells overexpressing TFPIα (A and C) or TFPIβ (B and D) were validated by Taqman single assays and qRT-PCR. Results were normalized against the endogenous controls PMM1 and U6 snRNA and the relative expression calculated using the comparative Ct method. Values are presented as mean (n = 3) fold change (FC) of empty vector pTOPO control + SEM of three biological replicates. White bars indicate array expression values, black bars represent Taqman verification values. Dotted lines indicate FC  = |2|.

**Figure 4:**
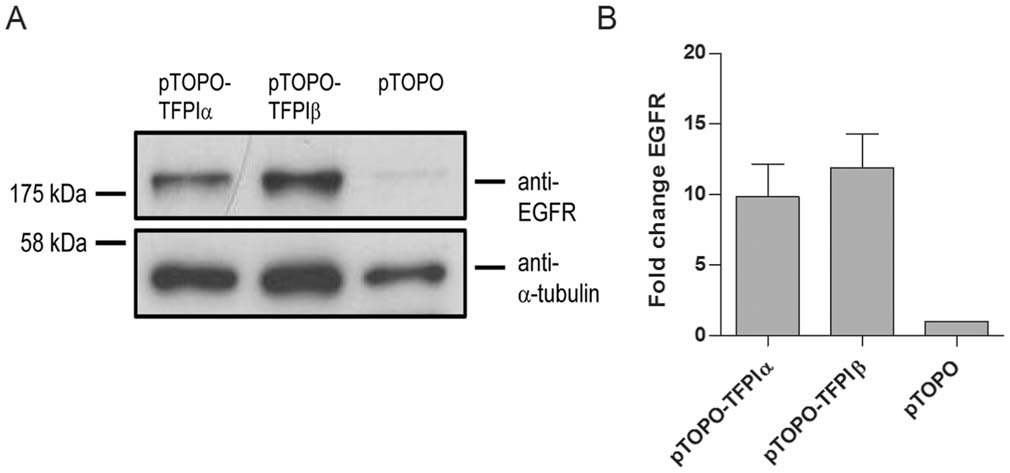
EGFR protein levels detected in the lysate of transfected SK-BR-3 cells by Western blot analysis. Proteins were separated on a SDS-polyacrylamide gel, transferred to a nitrocellulose membrane and detected using an anti-EGFR antibody. Anti-actin was used as a protein loading control. (A) Western blot of one representative experiment. (B) Quantification of three independent experiments using ImageJ (n = 3+ SEM).

To validate the results obtained from the microarray and miRNA array analyses, qRT-PCR was performed on selected genes using Taqman single assays. 16 mRNAs, which were either among the most differentially expressed genes or identified as a key gene in the functional and/or cluster analyses, and 13 miRNAs, which were identified in the functional analysis or had predicted mRNA targets differentially expressed, were selected. All of the 16 mRNAs and 11 of the 13 miRNAs selected for verification were validated (Figure 3). The two miRNAs that were not validated, miR-103 and miR-652, had expression values just beneath the fold change limit of |2|. The correlation between the array and qRT-PCR values was significant for both mRNA (r  =  0.9, *P*<.0001) and miRNA (r  =  0.8, *P*<.0001) measurements. The fold change values were generally higher when measured by qRT-PCR than in arrays, which is consistent with earlier reports [19]. Increased mRNA expression of *EGFR* was observed in both cell lines overexpressing TFPIα or TFPIβ, and it was identified as a key molecule in the functional and network analyses, in connection to both Cancer and Inflammation. EGFR protein levels were validated in the cell lysates by Western blotting, and the results showed elevated EGFR protein expression in both cell lines overexpressing TFPI (Figure 4). The mRNA and protein levels corresponded as higher EGFR levels were measured in cells overexpressing TFPIβ than in cells overexpressing TFPIα by either method.

## Discussion

We recently reported that stable overexpression of TFPIα or TFPIβ induced death receptor activated apoptosis and inhibited proliferation of SK-BR-3 breast cancer cells [18]. The molecular basis of the anti-tumor effects are, however, still unclear. To further investigate the cellular processes affected by TFPI and the possible molecular mechanisms behind the effects observed we characterized the global mRNA and miRNA expression profiles of these SK-BR-3 cell lines. To our knowledge, this is the first report on mRNA and miRNA profiles in cancer cells overexpressing TFPI. A total of 242 and 801 mRNAs were differentially expressed in cells overexpressing TFPIα or TFPIβ, respectively. Notably, TFPI was not among the upregulated genes. This was because the primers that were used to amplify TFPI cDNA only recognized the coding regions, thus leaving out the 5'- and 3'-UTR. The overexpressed TFPIα and TFPIβ were therefore not detected by the probes on the array that recognized these UTR sequences of the TFPI mRNA transcripts. When the PLIER algorithm, which leaves out probes with low signal intensities, was used to calculate expression differences, TFPI was upregulated 8.9 fold in cells overexpressing TFPIα and 11.9 fold in cells overexpressing TFPIβ.

In line with our previous findings [18], several of the differentially expressed genes were found to be involved in apoptosis and proliferation. Furthermore, genes involved in cellular processes such as adhesion, migration, invasion and colony formation were differentially expressed, indicating additional growth-related effects of either isoform of TFPI in the cancer cells. This finding is in line with the increased metastatic growth we recently observed in MDA-MB-231 and Sum102 breast cancer cells after downregulation of TFPI [20], and provides possible candidate genes responsible for these functional effects. In addition to cancer cell development, the functional and network analyses revealed that many of the differentially expressed genes were also involved in the immune response. An association between cancer progression and the immune system is recognized [21] and chronic inflammation increases the risk of cancer development. Inflammatory molecules such as chemokines have been shown to regulate tumor development [22], and death receptor signaling may activate inflammation in addition to apoptosis [23]. Moreover, Van Den Boogaard and colleagues have previously linked coagulation and inflammation as they reported of an anti-inflammatory effect of recombinant (r) TFPIα in mice [24], supporting our findings.

Overexpression of either isoform of TFPI resulted in expression profiles that associated significantly with tumor grade and ER status in a breast cancer patient material. This indicates a possibly therapeutic potential of TFPI that could affect tumor grade and perhaps patient outcome. However, TFPIα and TFPIβ expression in the patient material was not associated with tumor grade or ER status illustrating TFPI as a poor predictive marker for these variables. The association between TFPI and ER status is also intriguing as anti-estrogen therapy is important in breast cancer treatment [25]. Overexpression of TFPIα or TFPIβ resulted in FC values of −1.6 and −4.5, respectively, for the ER gene. Preliminary results from our laboratory indicate that estrogen decreases TFPI expression in ER positive breast cancer cells, which could contribute to the estrogen-driven growth of such tumors. It is clear that tumor cells differs considerably from cell lines in the environment that they are growing, and the generalizability of results obtained from cell lines should be taken under consideration. However, studies comparing breast tumors with established tumor derived breast cancer cell lines have concluded that the cell lines mirror the primary tumors with high fidelity regarding subtype and genomic features [26], [27].

In a previous study, 16 genes were reported to be differentially expressed in HUVECs after treatment with rTFPIα [28]. None of these were among the 242 genes differentially expressed in cells overexpressing TFPIα. The reason for this discrepancy could be differences in the signal transduction mechanisms mediated by rTFPIα compared to endogenously expressed TFPI or differences between the cell types. The authors suggested that binding of rTFPIα to the very low density lipoprotein receptor (VLDLR) resulted in the downstream gene regulation. In contrast, our results indicated a possible involvement of the EGFR signal transduction pathway. This signaling pathway is initiated by growth factors such as EGF and TGFα and activates signal transduction through small GTPases, MAP kinase cascades (MAPKs), such as ERK, p38, and JNK, and the NF-κB, PI3K/Akt, and JAK/STAT pathways among others. Although EGFR signaling is usually associated with increased cell survival and growth [29], reports have shown that EGFR stimulation can have the opposite effect [30]–[35], particularly when at high concentrations [36]–[38]. The SK-BR-3 cells used in this study have a high endogenous expression of the EGFR. In addition to increased EGFR expression, we observed an increase in NF-κB [18] and Elk-1 transcription factor activity, but not AP1 activity, in cells overexpressing TFPIα or TFPIβ. Elk-1 and AP1 are downstream transcription factors activated by the MAPK signaling cascade. They are mainly regulated by the MAPKs ERK and JNK, respectively, thus indicating involvement of the MAPK ERK but not JNK. Moreover, increased levels of phosphorylated Akt were observed in cells overexpressing TFPI. These findings further indicate involvement of the EGFR pathway. rTFPIα has previously been shown to affect MAPK/ERK signaling, although this was in HUVECs and a decrease in signaling was seen [39].

miRNAs play an important role in cancer development as they can alter the expression of many tumor suppressor- and oncogenes [5]. Overexpression of either isoform of TFPI resulted in altered expression of several miRNAs, and the integrated miRNA-mRNA analysis showed that up to 32% of the differentially expressed mRNAs may have been regulated by miRNAs. Of the 26 miRNAs that had predicted target genes oppositely regulated when either isoform was overexpressed, 21 (miR-15a, miR-21, miR-23b, miR-29c, miR-30c, miR-93, miR-98, miR-101, miR-135a, miR-135b, miR-148b, miR-193b, miR-200a, miR-200b, miR-203, miR-205, miR-218, miR-339, miR-365, miR-429, and miR-454) have previously been linked to breast cancer [40]–[49]. Among the predicted target genes identified as differentially expressed in cells overexpressing TFPIα or TFPIβ were several of the key genes, which were involved in apoptosis (*ACSL1*, *STAT3*, *SWAP70*, *TPD52*, and *WNT5A*), proliferation (*ACSL1*, *STAT3*, *PDGFD*, *TPD52*, and *WNT5A*), cell movement (*ACSL1*, *FERMT2*, *NAV1*, *NT5E*, *PDGFD*, *STAT3*, *SWAP70*, and *WNT5A*), cancer (*ACSL1*, *FERMT2*, *NT5E*, *NTN4*, *PDGFD*, *TPD52*, *STAT3*, and *WNT5A*) and inflammation (*ACSL1*, *KIAA1217*, *PDGFD*, *TPD52*, *STAT3*, *SWAP70*, and *WNT5A*).

Interestingly, only a portion of the differentially expressed mRNAs and miRNAs were identical after overexpression of TFPIα or TFPIβ. It therefore seems likely that these genes were responsible for the effects observed in both cell lines related to cancer cell growth. However, additional mechanisms and processes were also affected, which were unique for each of the isoforms of TFPI. Compared to cells overexpressing TFPIα, more genes were differentially expressed in cells overexpressing TFPIβ, and processes such as Carbohydrate metabolism, Free Radical Scavenging, Lipid Metabolism, Cellular Response to Therapeutics, Drug Metabolism, Vitamin and Mineral Metabolism, and Amino Acid Metabolism were significantly affected (Table S3). This may indicate a specific effect of TFPIβ on the general metabolism of the cells. Furthermore, the association between the TFPIβ expression profile and relapse-free survival could indicate additional therapeutic potential of this isoform.

In summary, we here report the mRNA and miRNA expression profiles of SK-BR-3 breast cancer cells overexpressing TFPIα or TFPIβ. The differentially expressed genes provide a genetic insight into the pro-apoptotic and anti-proliferative effects previously observed in these cells [18]. In addition, our findings show that increased expression of either isoform of TFPI affected the expression of genes involved in the immune response and development of cancer, linking these processes. Moreover, the mRNA expression signatures correlated significantly with tumor grade and ER status, showing clinical relevance of the TFPI induced gene expression. The molecular mechanisms mediating the effects of TFPI are presently unknown, but functional analysis and Western blot analysis indicated that the EGFR signaling pathway could be involved.

## Supporting Information

Figure S1 **Network analysis of differentially expressed genes in cells overexpressing TFPIα compared to empty vector control cells.** The most significant network (z score  = 38, Fisher's exact test) which integrated both molecular function and disease is shown. Upregulated genes are red, downregulated genes are green. Genes with no color are not differentially expressed with the criteria set (FDR 5%, *p*-value <0.05 and fold change |2|). Gray lines show biological interaction between genes as identified by the software (whole lines  =  direct interaction, dotted lines  =  indirect interaction, arrows  =  act on). Blue lines show genes involved in inflammatory response, pink lines show genes involved in cell death.
(TIF)

Figure S2 **Network analysis of differentially expressed genes in cells overexpressing TFPIβ compared to empty vector control cells.** The most significant network (z score  = 37, Fisher's exact test) which integrated both molecular function and disease is shown. Upregulated genes are red, downregulated genes are green. Genes with no color are not differentially expressed with the criteria set (FDR 5%, *p*-value <0.05 and fold change |2|). Gray lines show biological interaction between genes as identified by the software (whole lines  =  direct interaction, dotted lines  =  indirect interaction, arrows  =  act on). Blue lines show genes involved in inflammatory disease, pink lines show genes involved in cellular growth and proliferation.
(TIF)

Table S1 Differentially expressed mRNAs following increased expression of TFPIα in SK-BR-3 cells.
(XLS)

Table S2 Differentially expressed mRNAs following increased expression of TFPIβ in SK-BR-3 cells.
(XLS)

Table S3 Functional annotation of differentially expressed mRNAs following overexpression of TFPIα or TFPIβ. All the significant categories involved in molecular and cellular function and diseases and disorders as annotated by the Ingenuity Pathway Analysis software are listed.
(XLS)

Table S4 Network analysis of genes differentially expressed in SK-BR-3 cells overexpressing TFPIα or TFPIβ compared to empty vector control cells (pTOPO). Genes in bold are members of the network which are differentially expressed.
(XLS)

Table S5 Differentially expressed miRNAs following overexpression of TFPIα or TFPIβ in SK-BR-3 cells.
(XLS)

Table S6 Functional annotation of differentially expressed miRNAs following overexpression of TFPIα or TFPIβ. The ten most the significant categories involved in molecular and cellular function and diseases and disorders as annotated by the Ingenuity Pathway Analysis software are listed.
(XLS)

Table S7 Differentially expressed miRNAs with predicted gene targets oppositely expressed at the mRNA level in SK-BR-3 cells overexpressing TFPIα. Predicted mRNA targets in bold were also identified as possible targets in cells overexpressing TFPIβ.
(XLS)

Table S8 Differentially expressed miRNAs with predicted gene targets oppositely expressed at the mRNA level in SK-BR-3 cells overexpressing TFPIβ. Predicted mRNA targets in bold were also identified as possible targets in cells overexpressing TFPIα.
(XLS)
