# Supplementary figures and images for: TFPI Alpha and Beta Regulate mRNAs and microRNAs Involved in Cancer Biology and in the Immune System in Breast Cancer Cells
Source: PLoS One. 2012 Oct 5;7(10):e47184. doi: 10.1371/journal.pone.0047184 (PMC3465304; doi:10.1371/journal.pone.0047184)

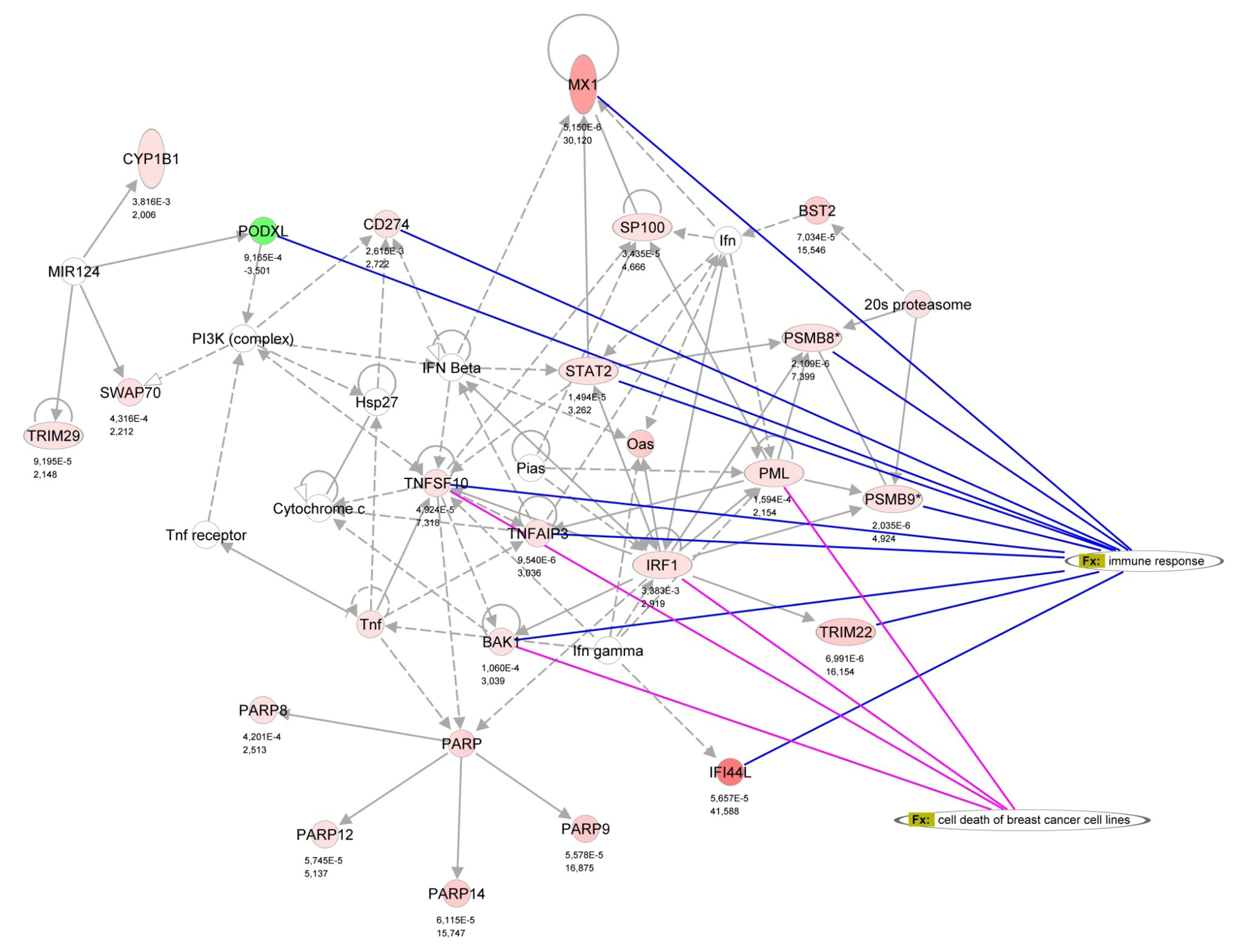

Supplement: Figure S1 — Network analysis of differentially expressed genes in cells overexpressing TFPIα compared to empty vector control cells. The most significant network (z score = 38, Fisher's exact test) which integrated both molecular function and disease is shown. Upregulated genes are red, downregulated genes are green. Genes with no color are not differentially expressed with the criteria set (FDR 5%, p-value <0.05 and fold change |2|). Gray lines show biological interaction between genes as identified by the software (whole lines = direct interaction, dotted lines = indirect interaction, arrows = act on). Blue lines show genes involved in inflammatory response, pink lines show genes involved in cell death. (TIF) [file pone.0047184.s001.tif]

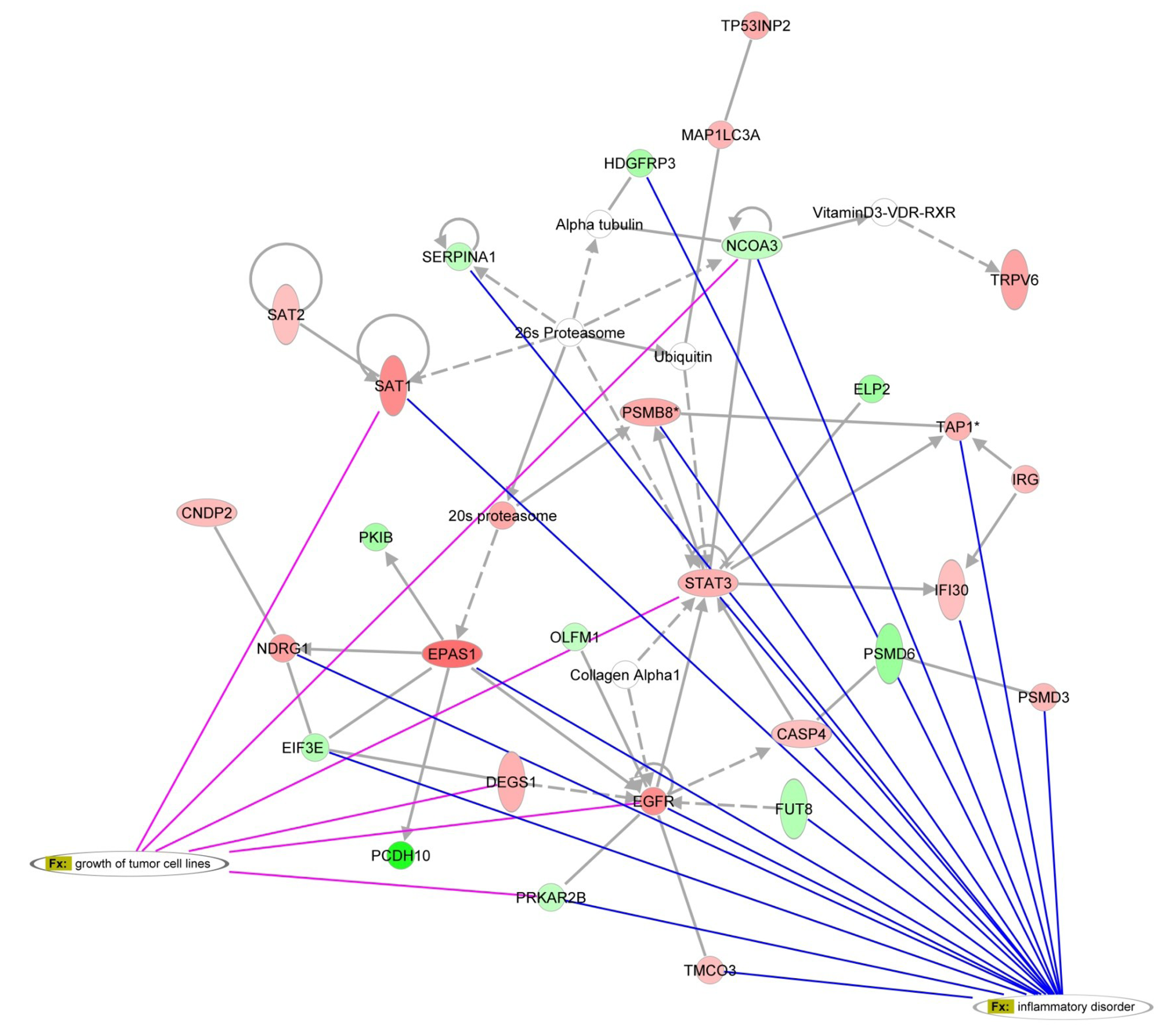

Supplement: Figure S2 — Network analysis of differentially expressed genes in cells overexpressing TFPIβ compared to empty vector control cells. The most significant network (z score = 37, Fisher's exact test) which integrated both molecular function and disease is shown. Upregulated genes are red, downregulated genes are green. Genes with no color are not differentially expressed with the criteria set (FDR 5%, p-value <0.05 and fold change |2|). Gray lines show biological interaction between genes as identified by the software (whole lines = direct interaction, dotted lines = indirect interaction, arrows = act on). Blue lines show genes involved in inflammatory disease, pink lines show genes involved in cellular growth and proliferation. (TIF) [file pone.0047184.s002.tif]
